# A Remarkable New Species of *Liparis* (Orchidaceae) from China and Its Phylogenetic Implications

**DOI:** 10.1371/journal.pone.0078112

**Published:** 2013-11-13

**Authors:** Lin Li, Haifei Yan

**Affiliations:** 1 Key Laboratory of Plant Resources Conservation and Sustainable Utilization, South China Botanical Garden, Chinese Academy of Sciences, Guangzhou, China; Montreal Botanical Garden, Canada

## Abstract

In the present study, we formally describe *Liparis pingxiangensis* as a new species from Guangxi, China on the basis of morphological and molecular phylogenetic analyses. It is easily distinguished from closely related species by strongly curved column without column wings, and broadly rhombic-elliptic lip with 2 uncinate calli at the base. In particular, it differs most markedly from its congeners in possessing two pollinia attached by long and prominent caudicles (not stipes), to a distinct sticky disc. This type of pollinarium, as far as we know, is not found in any other species of *Liparis*, and is also unique among the orchids with waxy pollinia. We then proceeded to a phylogenetic analysis to ascertain the systematic position of this enigmatic species. Molecular study based on nuclear ribosomal ITS and plastid *mat*K DNA sequence data supports *L. pingxiangensis* as a distinct species, which forms an independent lineage sister to *L. nervosa* and its allies (93% BS, 1.00 BPP). In the light of previous work, the findings have important implications for a better understanding of the well-supported pattern mainly based on vegetative features in Malaxideae.

## Introduction

As currently understood, the genus *Liparis* Rich. in the tribe Malaxideae (Epidendroideae, Orchidaceae) [1] is relatively large and cosmopolitan, consisting of approximately 350 species, with the majority occurring in tropical and subtropical regions, with some representatives extending to the temperate regions [2], [3], [4]. *Liparis* species are terrestrial, epiphytic or rarely lithophytic. Generally, it is readily characterized by floral morphology, such as resupinate flowers which usually have narrow linear petals; 2-parted lip (forming the hypochile and the epichile), most common bearing a basal bilobed callus; elongate, slender, curved, and slightly winged column lacking a foot [3], [4], [5].

A previous molecular phylogenetic study indicated that *Liparis* in the traditional broad sense is polyphyletic [6]. The taxonomy of the genus is complicated and controversial. In spite of clear and distinct differences, *Liparis* has been divided differently by different authors in the past [7], [8], [9], [10], [11]. Some clades are well resolved and can be diagnosed morphologically, others less so, requiring more data to see clear evidence of interrelationships among the tribe [1]. Before the establishment of the robust classification schemes, the traditional recognition of *Liparis* s.lato is employed in this paper.

In China, the genus *Liparis* is represented by approximately 63 species (20 endemic) [12]. During a recent field trip to Guangxi, China, a terrestrial species was collected by one of the authors. At first glance the plants appeared to resemble the ordinary *Liparis* species, but a closer study revealed that its most striking feature was the pollinarium structure, which we had never seen before. This unusual specimen has two specialized waxy pollinia, with two long and prominent caudicles, attached to a terminal viscidium. Another unusual character is its column, which is strongly curved without column wings. Molecular phylogenetic study using nuclear ribosomal ITS and plastid *mat*K sequences was initiated to ascertain its systematic position. Our thorough morphological comparison and phylogenetic analysis indicated that it represents an unknown species, which is described and illustrated here for the first time.

## Materials and Methods

### Ethics statement

The collecting location reported in this work is not protected in any way. The species collected here are currently not included in the Chinese Red Data Book. No specific permits are required for the present study.

### Nomenclature

The electronic version of this article in Portable Document Format (PDF) in a work with an ISSN or ISBN will represent a published work according to the International Code of Nomenclature for algae, fungi, and plants, and hence the new names contained in the electronic publication of a PLOS ONE article are effectively published under that Code from the electronic edition alone, so there is no longer any need to provide printed copies.

In addition, new names contained in this work have been submitted to IPNI, from where they will be made available to the Global Names Index. The IPNI LSIDs can be resolved and the associated information viewed through any standard web browser by appending the LSID contained in this publication to the prefix http://ipni.org/. The online version of this work is archived and available from the following digital repositories: PubMed Central, LOCKSS.

### Morphological observations

Morphological description of the new species was based on examination of fresh and pressed specimens. Details of the flowers, particularly the gynostemium and pollinarium were examined and photographed under a stereomicroscope (Olympus MD-90). The morphological comparison with other species of Malaxideae was based on study of live plants in the field and in cultivation, herbarium specimens, and information gathered in the literature searches. The specimens examined have been deposited in the herbarium of South China Botanical Garden, Chinese Academy of Sciences (IBSC).

### Taxon sampling

We downloaded 74 sequences for 54 species from GenBank. In addition, we sequenced four species including three samples of the new taxon for this work. The ingroup included a total of 80 sequences of 58 species, representing all of the major clades in the tribe Malaxideae sensu Pridgeon et al. [1]. Three representative species of *Acanthephippium* Bl. ex Endl., *Collabium* Bl., and *Eria* Lindl. were selected as outgroups following previous phylogenetic studies [13]. GenBank accession numbers for all the DNA sequences and voucher information are given in Table S1.

### Molecular markers

We analyzed the nucleotide sequences of the nuclear ribosomal internal transcribed spacers (nrITS), as well as the plastid *mat*K, which have demonstrated their utility for inferring phylogenetic relationships at various taxonomic levels among the Malaxideae and other members in Orchidaceae [6], [14], [15], [16], [17]. Extraction, amplification and sequencing of genomic DNA were carried out from fresh leaf tissue using the modified 2× CTAB method after leaf tissue was ground in liquid nitrogen [18]. The nrITS region (ITS1-5.8S-ITS2) was amplified via a polymerase chain reaction (PCR) with the primers 17SE and 26SE of Sun et al. [19]. The *mat*K gene region was amplified and sequenced using the primers as described by Whitten et al. [20] and Cameron [6].

### Sequence alignment and phylogenetic analysis

DNA sequences were initially aligned with the computer program CLUSTAL X version 1.83 [21] with minor subsequent manual adjustment using the software Se-Al verion 2.0a11 [22]. To evaluate congruence between different DNA regions, we analyzed each dataset (nrITS and *mat*K) separately to see if they produced a similar topology. We also conducted the incongruence length difference (ILD) test [23] with 500 replicates of the heuristic search in PAUP* verion 4.0b10 [24]. The 1% level of significance was chosen as described in [25]. The ILD value in this study was 0.23, suggesting congruence between the two regions. We therefore analyzed these two datasets in combination.

Separate and combined phylogenetic analyses were carried out using maximum parsimony (MP) and Bayesian inference (BI) as implemented in PAUP* [24] and MrBayes verion 3.1.2 [26], respectively. MP analyses were performed using heuristic searches with 1,000 replicates of random taxon addition, and tree rearrangements using tree bisection-reconnection (TBR) branch swapping, and MULTREES option in effect, and simple addition and ACCTRAN optimization. All characters were treated as unordered and had equal weights, gaps considered as missing data. Internal support for clades was assessed by non-parametric bootstrapping (BS) with 1,000 bootstrap replicates [27].

The best-fitting model of sequence evolution in Bayesian analyses was chosen for each marker using ModelTest 3.7 [28]. The Akaike information criterion was implemented. Evolutionary models that best fitted the ITS and *mat*K were GTR+I+G and TVM+I+G, respectively. BI analyses settings were: the Markov chain Monte Carlo (MCMC) algorithm was run for 2 million generations, and sampled every 200 generations. A conservative burn-in (25%) was applied after checking for stability on the log-likelihood curves and split variances being <0.01. A majority rule consensus tree was calculated from the remaining samples. Branch support was determined by Bayesian Posterior Probabilities (BPP).

## Results

### Phylogenetic analysis

**Figure 1:**
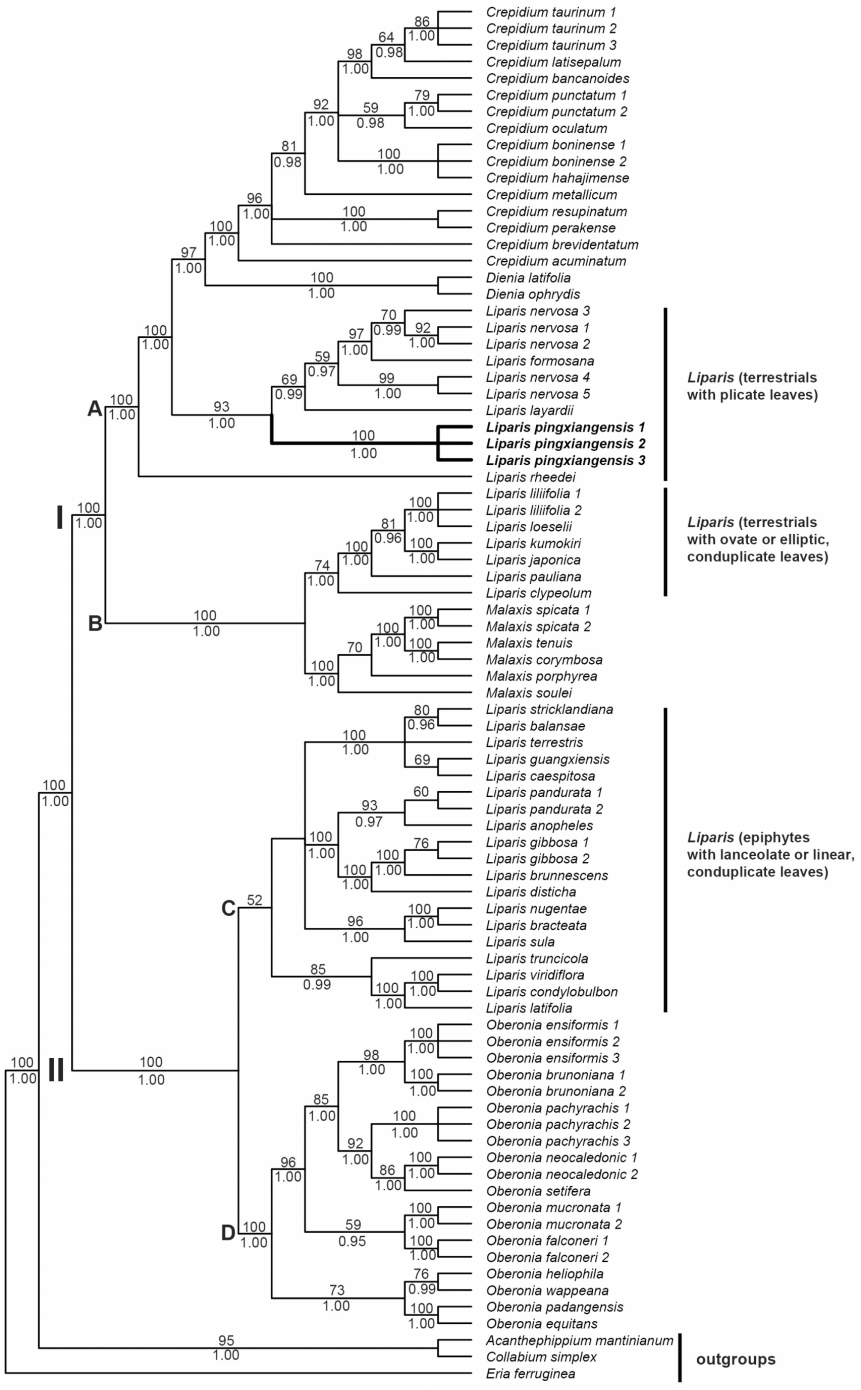
Phylogenetic tree. Strict consensus tree of the 1932 most parsimonious trees obtained from sequences of the combined nrITS and plastid *mat*K DNA sequence data (2280 steps, CI = 0.53, RI = 0.89), showing the position of *Liparis pingxiangensis*. Parsimony bootstrap proportions higher than 50% and Bayesian posterior probabilities (BPP) more than 0.95 are shown above and below branches, respectively.

The length of aligned matrix of nrITS was 970 bp, of which 516 (53%) were variable and 410 (42%) were parsimony-informative. The aligned *mat*K matrix consisted of 1470 characters, of which 409 (28%) were variable and 266 (18%) were parsimony-informative. There was no significant difference in topographies between nrITS and *mat*K (see Figure S1). For the combined data, the heuristic search found 1932 MPTs with a length of 2280 steps, consistency index (excluding uninformative characters) = 0.53 and retention index = 0.89. The topologies from the MP and Bayesian analyses were congruent. The strict consensus tree based on combined data (with BS and BPP) is shown in Figure 1.

### Taxonomic treatment

#### Liparis pingxiangensis

**Figure 2:**
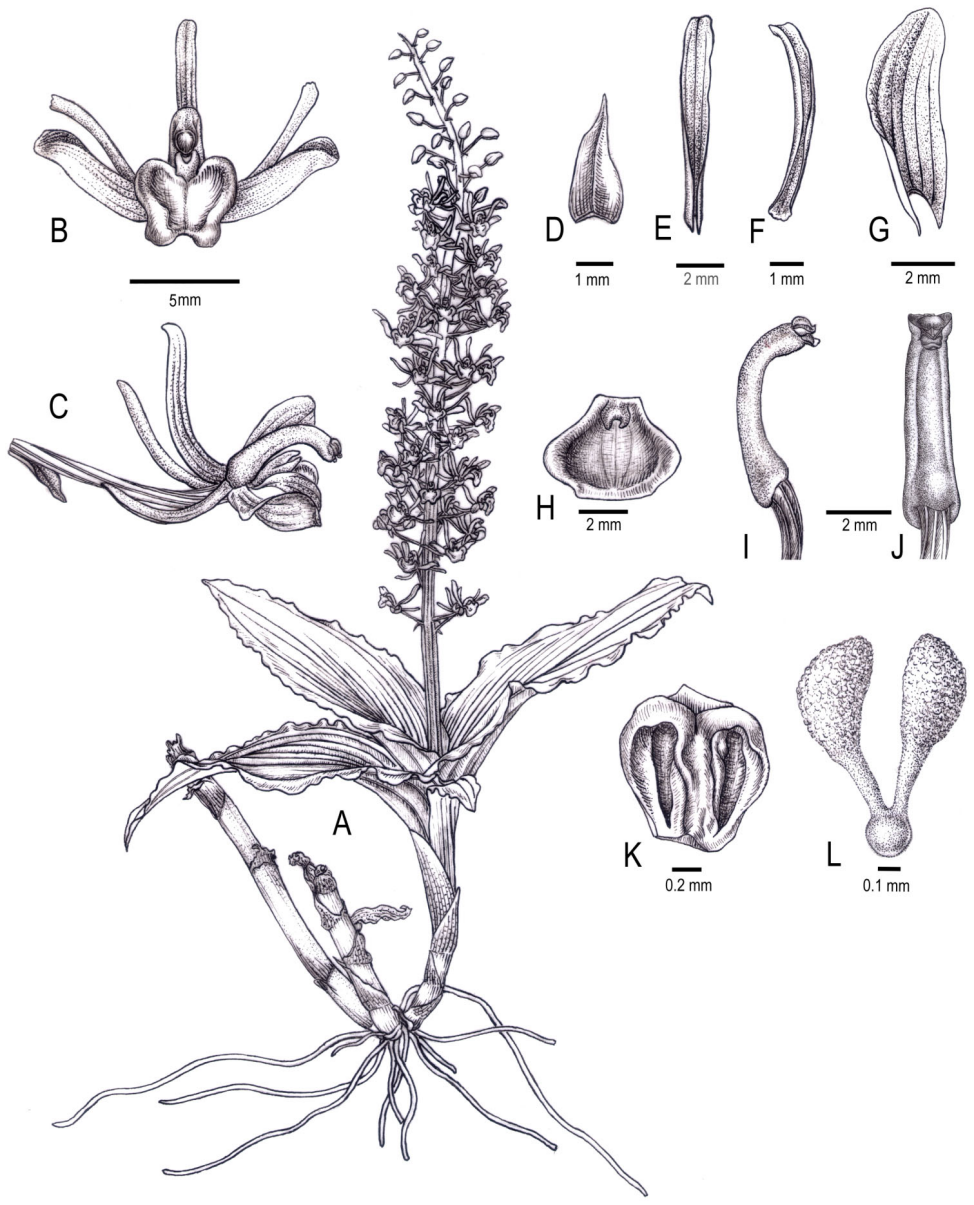
Liparis pingxiangensis. A. habit, B. flower, frontal view, C. flower, lateral view, D. bract, E. dorsal sepal, F. petal, G. lateral sepal, H. lip, I. column, lateral view, J. column without anther cap, ventral view, K. anther cap, L. pollinarium. Drawn by Yun-Xiao Liu.

**Figure 3:**
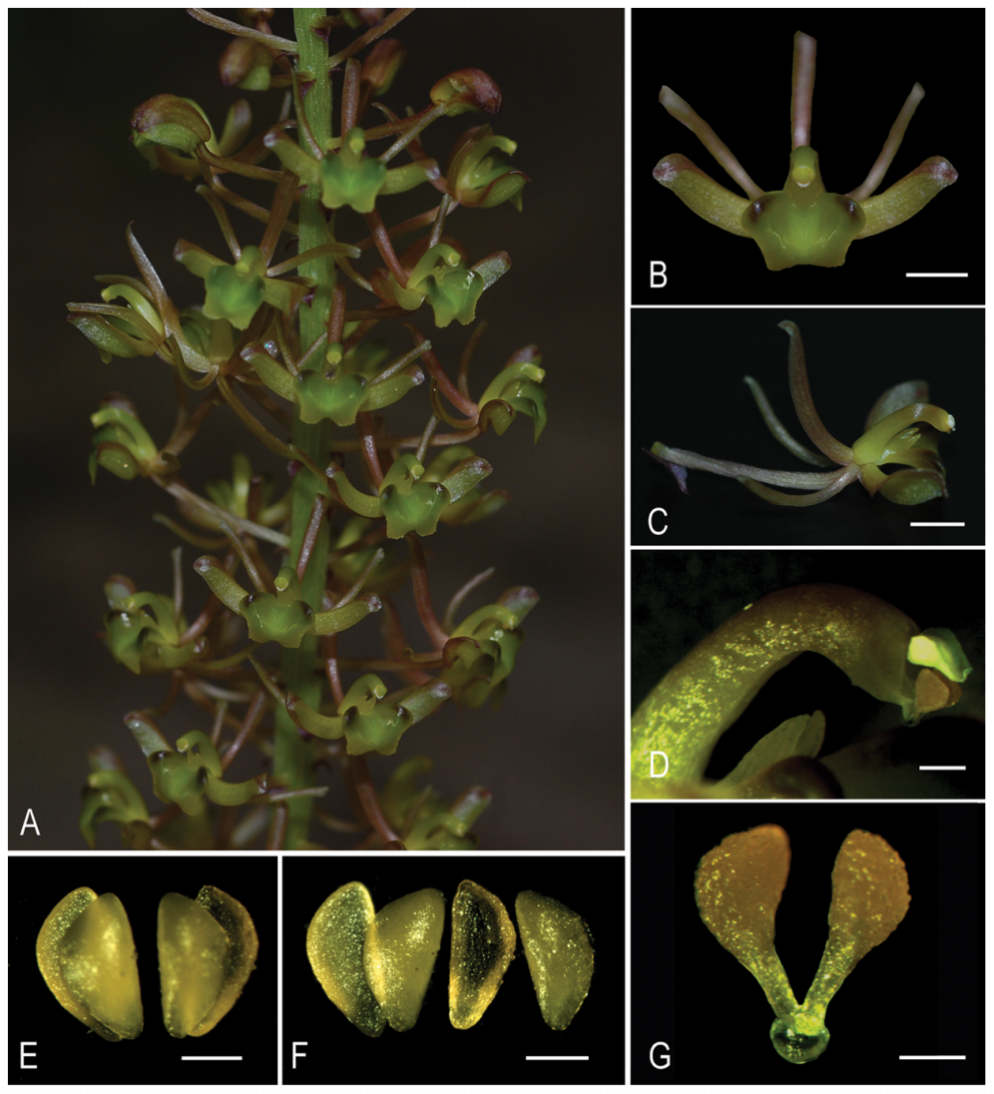
Morphology of *Liparis pingxiangensis* (A–D, G) and allied species *L. nervosa* (E–F). A. inflorescence, B. flower, front view, C. flower, lateral view, D. gynostemium including anther, clinandrium and viscidium attached to apex of rostellum, lateral view, E. two pairs of close pollinia, F. two pairs of separate pollinia, G. pollinarium including pollinia, caudicles and viscidium. Scale bars, 3(B–C); 1 mm (D); 0.3 mm (E–G).

L. Li & H. F. Yan, sp. nov. [urn:lsid:ipni.org: names: 77131431-1] (Figs. 2, 3A–D, G). Type: — CHINA. Guangxi: Pingxiang, mixed deciduous forests, terrestrial in moist and shady grassy slopes, rare, collected 23 June 2011, flowered and pressed from plant cultivated in an experimental greenhouse of SCBG, 2 Apr 2012, *L. Li 151* (HOLOTYPE: IBSC).

Species affinis *L. nervosae* (Thunb.) Lindl., a qua labiis late rhombico-ellipticus, callis uncinnatis praeditis, columnis arcuatis sine alis, polliniis duobus caudiculis longis affixis cum viscidio magno recedit.

#### Terrestrial herb, up to 20 cm in height

Pseudobulbs cylindric, tapering, enveloped by 3–4 clasping basal foliaceous sheaths that fall away and bares the bulbs before the new growth arises; old leafless pseudobulbs usually covered with several membranous sheaths at internodes, 8–10 cm long, 2.5–3 cm in diameter. Leaves 3–4, basal, amplexicaul, blade elliptic-lanceolate to ovate-elliptic, plicate, membranous or herbaceous, base slightly oblique and contracted into petioles, margin wavy or undulating, apex acute, 14–16 cm long, 5–8 cm wide; petiole sheathlike, 3–5 cm long, not articulate. Inflorescence terminal, racemose, 24–33 cm long; peduncle ridged, ebracteate; rachis 16–20 cm with many well-spaced flowers. Floral bracts ovate-triangular, carinate on the abaxial surface, dark purple, 3–3.5 mm long. Ovary ridged and grooved, greenish brown, 1.0–1.3 cm long. Flowers resupinate, spreading, 1.2–1.3 cm across, yellowish-green with dull purple markings, column greenish-yellow. Dorsal sepal broadly linear-oblong, 3-veined, 8–8.5 mm long, 1.3–1.5 mm wide, obtuse at apex, often recurved or reflexed, margins revolute, dull purple; lateral sepals broader than the dorsal sepal, ovate-elliptic, slightly oblique, 6–7 mm long, 2–2.5 mm wide, 5-veined, obtuse at apex, recurved or reflexed, upside dull purple, lower side greenish yellow. Petals linear, more or less falcate, 7.5–8 mm long, 0.7–0.9 mm wide, 1-veined, margins revolute, brownish purple, tinged with greenish yellow. Lip entire, broadly rhomboid-elliptic, slightly fleshy, concave in the middle, base abruptly contracted, with a pair of subulate, somewhat uncinate calli, curved downward or reflexed back near the middle, apex emarginate, margin slightly erose, greenish yellow, with conspicuous dark brownish blotch on each side, 4.0–4.2 long, 4.5–5.6 mm wide. Column elongated, strongly incurved or arcuate near apex, 5–5.8 mm long, column wings absent. Stigma transverse, concave, subquadrate. Rostellum protruding, approximately triangular, apex obtuse, whitish. Anther terminal, 2-celled, persistent, oblong-ovoid, ca. 1.0 mm long. Pollinarium ca. 1.0 mm long, formed by 2 pollinia, more or less, but not completely cleft, elongate-obovoid, bilaterally flattened, somewhat club-shaped, hard, waxy, darker yellow or orange, provided with two distinctly long caudicles, ca. 0.3 mm long, hyaline, with light yellow tinges, attached to the rostellum by a terminal, rounded viscidium, white to transparent, thick.

#### Distribution, habitat and ecology

*Liparis pingxiangensis* is terrestrial, forming more or less scattered colonies on shady and damp areas with small ravines, in wet to most soils and humus, on the steeper slopes, at elevations of around 800 m in the mixed deciduous forest of southwest China's Guangxi Zhuang Autonomous Region. Flowering occurs in early spring, from early until late April. Up to now, it has not been observed in fruits.

#### Conservation status

A rare species occurs in a rather small population (no more than 10 individuals). As far as we can currently observe, it is known only from the type collection and a neighboring population. The forest has been experiencing a continuing decline in quality of habitat due to deforestations. Using the World Conservation Union Red List Categories and Criteria [29], *L. pingxiangensis* should be treated as critically endangered due to its rarity and the threat of disturbance. More studies at the two nearby localities may shed light on this enigmatic species.

#### Etymology

The species is named after the site of its first discovery, Pingxiang, Guangxi province, China.

#### Species recognition

*Liparis pingxiangensis* is very distinctive among species in the genus. It is the only species with two pollinia, through distinctly long and prominent caudicles, attached to a thick viscidium (Figs. 2-L, 3-G). As we all know, this pollinarium type has not been discovered in any other group of orchids. It is morphologically similar to *L. nervosa* (Thunb.) Lindl., a widespread terrestrial species with plicate leaves, but it can be easily distinguished from the latter by having strongly curved column without column wings, broadly rhombic-elliptic lip with 2 uncinate calli at the base. Two pollinia are also found in *L. fissipetala* Finet, but this is an epiphytic plant with ovoid pseudobulbs, strongly crisped-margined leaves, deeply bilobed or Y-shaped petals, and two pollinia with much shorter caudicles, without a true viscidium [30].

Our molecular results based on nrITS and *mat*K DNA sequence data support *L. pingxiangensis* as a distinct species. It clearly and precisely indicates that the individuals of *L. pingxiangensis* form a monophyletic clade (100% BS, 1.00 BPP), being strongly supported as sister to *L. nervosa* and its allies (93% BS, 1.00 BPP).

## Discussion

Orchids are well known for specialized floral morphology. Most orchids (Orchidoideae and Epidendroideae) develop pollinia, which are often accompanied by appendages, known as pollinaria [31], [32]. The morphological features of pollinaria are taxonomically informative and have always been considered of taxonomic significance in Orchidaceae [33], [34].

Malaxideae has always been characterized by four ‘naked pollinia’ that are devoid of any typical associated appendages such as caudicles or stipes on their pollinia, although they appear to have tiny and semi-liquid viscidium in most cases [31], [32]. The discovery of true pollinia or pollinaria provides valuable insight into the morphological diversification of pollinia in Malaxideae.

The resulting cladograms comprehend two main clades I and II, which are further divided into A and B, C and D respectively. In both analyses (MP and Bayesian Inference), *Liparis* s.lato is found in three major clades (A–C). Overall, relationships agree with those recovered in the phylogenetic analyses of Cameron [6]. Clade A consisting of *Liparis* with distinctly plicate leaves, *Crepidium and Dienia* (formerly included in the genus Malaxis s.lato), are resolved as sister clades with strong support (100% BS, 1.00 BPP); clade B consists of two small clades. *Liparis* with ovate or elliptic, conduplicate leaves and *Malaxis* are strongly resolved as sister clades (100% BS, 1.00 BPP); clade C, a weakly supported group including epiphytic *Liparis* with lanceolate or linear, conduplicate leaves is resolved as sister (100% BS, 1.00 BPP) to clade D, a strongly supported monophyletic group (100%BS), comprising all currently recognized species of *Oberonia*. In other words, terrestrial *Liparis* have a very close relationship with *Malaxis* s.lato with similar habitat and leaves (clade I). Likewise, a close relationship of epiphytic *Liparis* between *Oberonia* is again presented based on extensive sample (clade II).

*L. pingxiangensis* has very special pollinaria, but even then it is still clearly positioned within the terrestrial clade, taxa with plicate leaves. Our analyses provide further insight into the well-supported pattern based first on vegetative features, instead of reproductive features that are usually emphasized in establishing orchid classification systems. The results confirm that the generic delimitations between *Liparis* and related genera need to be reevaluated. However, more detailed studies are required to allow for the precise delimitation of these groups and to clarify their relationships in Malaxideae.

## Supporting Information

Figure S1 **Comparison of phylogenetic trees.** Strict consensus trees generated from nrITS and matK DNA sequences, respectively. Numbers above branches indicate bootstrap values (BS) higher than 50%; asterisks below branches represent Bayesian posterior probabilities (BPP) more than 0.95.
(PDF)

Table S1 **Taxa analyzed, voucher information, and GenBank accession numbers for the DNA sequences.** Sequences generated in this study are marked with an asterisk (*).
(DOC)
