# Supplementary figures and images for: A Remarkable New Species of Liparis (Orchidaceae) from China and Its Phylogenetic Implications
Source: PLoS One. 2013 Nov 13;8(11):e78112. doi: 10.1371/journal.pone.0078112 (PMC3827247; doi:10.1371/journal.pone.0078112)

ITS

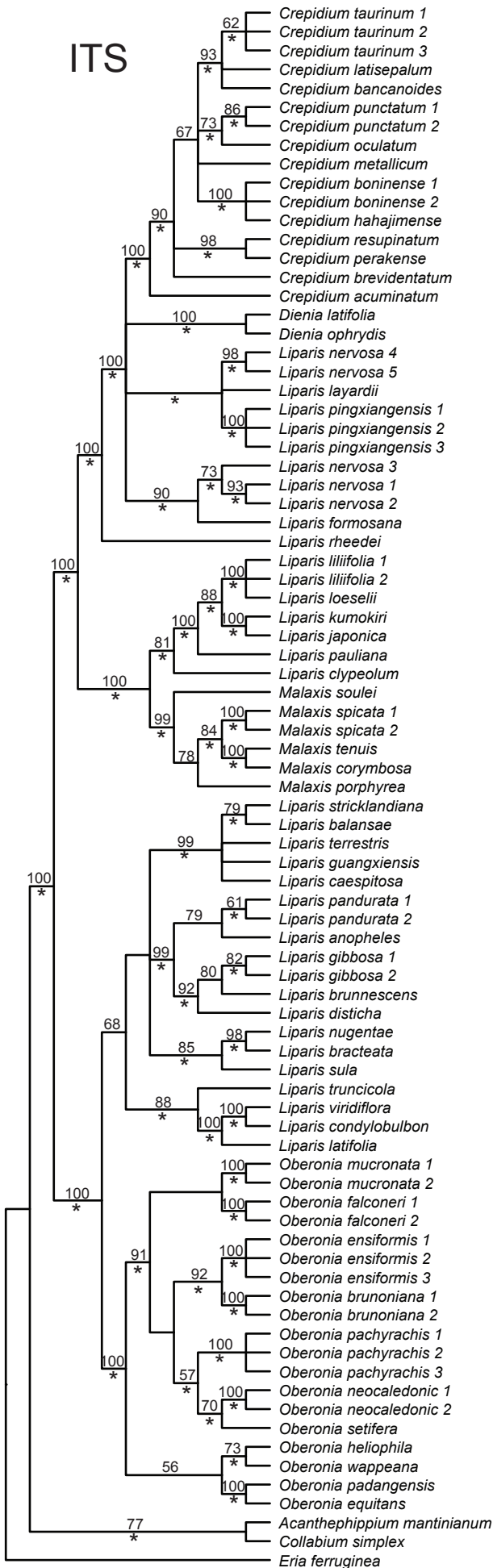

matK

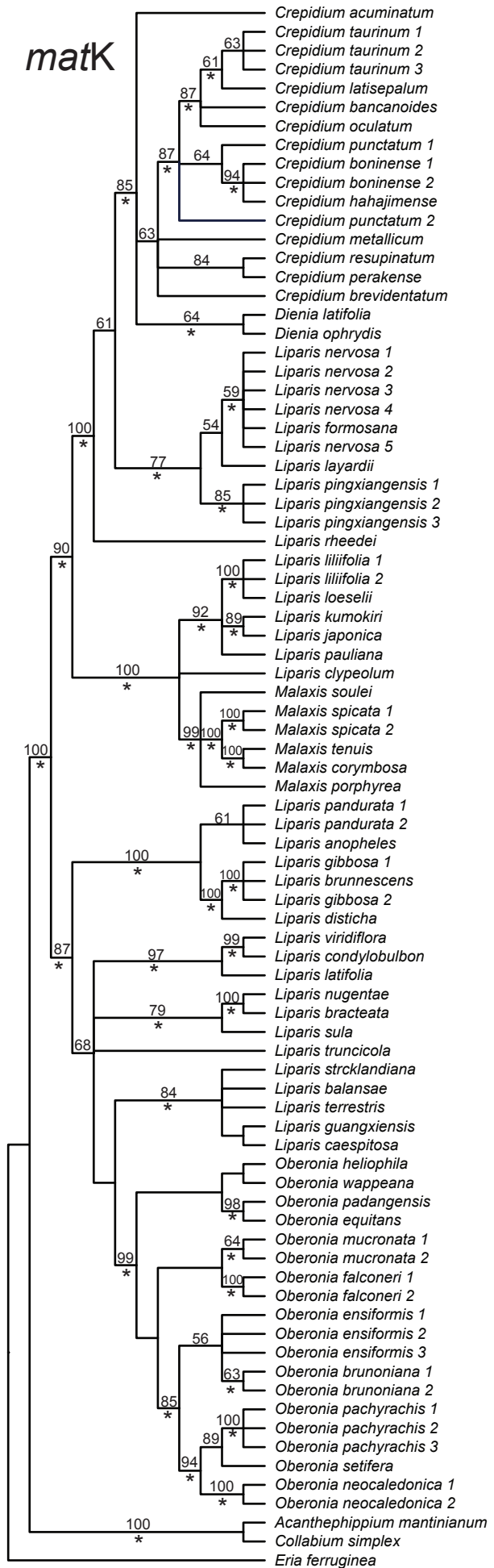

Supplement: Figure S1 — Comparison of phylogenetic trees. Strict consensus trees generated from nrITS and matK DNA sequences, respectively. Numbers above branches indicate bootstrap values (BS) higher than 50%; asterisks below branches represent Bayesian posterior probabilities (BPP) more than 0.95. (PDF) [file pone.0078112.s001.pdf]
