# Supplementary material for: A Remarkable New Species of Liparis (Orchidaceae) from China and Its Phylogenetic Implications
Source: PLoS One. 2013 Nov 13;8(11):e78112. doi: 10.1371/journal.pone.0078112 (PMC3827247; doi:10.1371/journal.pone.0078112)
Supplement: Table S1 — Taxa analyzed, voucher information, and GenBank accession numbers for the DNA sequences. Sequences generated in this study are marked with an asterisk (*). (DOC) [file pone.0078112.s002.doc]

**Table S1. Taxa analyzed, voucher information, and GenBank accession numbers for the DNA sequences. Sequences generated in this study are marked with an asterisk (*).**

| Species Name | Vouchers | ITS | *mat*K |
| --- | --- | --- | --- |
| *Acanthephippium mantinianum* | MWC397 | AF521081 | AF263618 |
| *Crepidium acuminatum* | M. Watanabe s. n. (TNS) | AB290884 | AB290892 |
| *C. bancanoides* | Yukawa 95-101 (TNS) | AB290885 | AB290893 |
| *C. boninense 1* | Kato 060006 (MAK) | AB290889 | AB290897 |
| *C. boninense 2* | Ohi-Toma s. n. (TI) | AB290887 | AB290895 |
| *C. brevidentatum* | Yukawa 97-2066 (TNS) | AB290886 | AB290894 |
| *C. hahajimense* | Ohi-Toma s. n. (TI) | AB290888 | AB290896 |
| *C. latisepalum* | T. Motley & K. Cameron 2254 (NY) | AY907112 | AY907178 |
| *C. metallicum* | Singapore B. G. cult 1522 | AY907113 | AY907180 |
| *C. oculatum* | Nazarudin s. n. (TNS) | AB290890 | AB290898 |
| *C. perakense* | Nazarudin s. n. (TNS) | AB290891 | AB290899 |
| *C. punctatum 1* | Leiden cult. 980113 | AY907117 | AY907184 |
| *C. punctatum 2* | Singapore B. G. cult. 2020 | AY907116 | AY907183 |
| *C. resupinatum* | K. Wood 9548 (NY) | AY907118 | AY907185 |
| *C. taurinum 1* | T. Motley & K. Cameron 2150 (NY) | AY907125 | AY907192 |
| *C. taurinum 2* | T. Motley & K. Cameron 2152 (NY) | AY907127 | AY907194 |
| *C. taurinum 3* | T. Motley & K. Cameron 2153 (NY) | AY907128 | AY907195 |
| *Collabium simplex* | AK991017/1/04 (Z) | EF670387 | EF670434 |
| *Dienia latifolia* | Leiden cult. 914208 | AY907111 | AY907177 |
| *D. ophrydis* | Singapore B. G. cult. 3380 | AY907114 | AY907181 |
| *Eria ferruginea* | MWC590 | AF521071 | AF263660 |
| *Liparis anopheles* | Leiden cult. 980165 | AY907075 | AY907139 |
| **L. balansae* | L. Li 152 (IBSC) | KF589874 | KF589880 |
| *L. bracteata* | P. Weston s.n. | AY907076 | AY907140 |
| *L. brunnescens* | Leiden cult. 20030224 | AY907098 | AY907165 |
| *L. caespitosa* | Leiden cult. 20030195 | AY907077 | AY907141 |
| *L. clypeolum* | J.-Y. Meyer 1029 (NY) | AY907079 | AY907143 |
| *L. condylobulbon* | Leiden cult. 20030654 | AY907080 | AY907144 |
| *L. disticha* | Leiden cult. 20010180 | AY907081 | AY907145 |
| *L. formosana* | K. Cameron 2151 (NY) | AY907082 | AY907147 |
| *L. gibbosa 1* | K. Cameron 2061 (NY) | AY907083 | AY907148 |
| *L. gibbosa 2* | K. Cameron 2211 (NY) | AY907084 | AY907149 |
| **L. guangxiensis* | L. Li 153 (IBSC) | KF589875 | KF589881 |
| *L. japonica* | EWH: Lee 102 | EU017406 | EU017429 |
| *L. kumokiri* | EWH: Lee 228 | EU017417 | EU017439 |
| *L. latifolia* | Singapore B. G. cult. 837 | AY907088 | AY907153 |
| *L. layardii* | K. Cameron 2060 (NY) | AY907089 | AY907155 |
| *L. liliifolia 1* | Chase O-214 (K) | AF521067 | AF263667 |
| *L. liliifolia 2* | EWH:Lee 316 | EU017427 | EU017449 |
| *L. loeselii* | B. Ewacha s.n. | AY907091 | AY907157 |
| *L. nervosa 1* | SBB-0080 | JN114604 | JN004492 |
| *L. nervosa 2* | SBB-0077 | JN114601 | JN004490 |
| *L. nervosa 3* | SBB-0075 | JN114599 | JN004488 |
| *L. nervosa 4* | SBB-0073 | JN114597 | JN004486 |
| *L. nervosa 5* | SBB-0071 | JN114595 | JN004484 |
| *L. nugentae* | P. Weston s.n. | AY907093 | AY907159 |
| *L. pandurata 1* | Leiden cult. 20020341 | AY907094 | AY907160 |
| *L. pandurata 2* | Singapore B. G. cult. 85 | AY907095 | AY907161 |
| *L. pauliana* | K. Cameron 2169 (NY) | AY907096 | AY907163 |
| **L. pingxiangensis 1* | L. Li 151 (IBSC) | KF589870 | KF589876 |
| **L. pingxiangensis 2* | L. Li 154 (IBSC) | KF589871 | KF589877 |
| **L. pingxiangensis 3* | L. Li 157 (IBSC) | KF589872 | KF589878 |
| *L. rheedei* | Leiden cult. 970454 | AY907097 | AY907164 |
| **L. stricklandiana* | L. Li 135 (IBSC) | KF589873 | KF589879 |
| *L. sula* | K. Cameron s.n. DNA#1174 | AY907104 | AY907171 |
| *L. terrestris* | Singapore B. G. cult. 3482 | AY907105 | AY907172 |
| *L. truncicola* | Leiden cult. 20030222 | AY907106 | AY907173 |
| *L. viridiflora* | NYBG cult. 2025 | AY907107 | AY907174 |
| *Malaxis corymbosa* | R. Coleman 1068 (AZ) | AY907110 | AY907176 |
| *M. porphyrea* | R. Coleman 226823 | AY907115 | AY907182 |
| *M. soulei* | R. Coleman 1069 (AZ) | AY907119 | AY907186 |
| *M. spicata 1* | MWC377 | AF521068 | AY368415 |
| *M. spicata 2* | C. McCartney s.n. | AY907124 | AY907191 |
| *M. tenuis* | R. Coleman 1019 (AZ) | AY907129 | AY907196 |
| *Oberonia brunoniana 1* | SBB-0795 | JN114625 | JN004519 |
| *O. brunoniana 2* | SBB-0786 | JN114622 | JN004515 |
| *O. ensiformis 1* | SBB-0798 | JN114628 | JN004522 |
| *O. ensiformis 2* | SBB-0796 | JN114626 | JN004520 |
| *O. ensiformis 3* | SBB-0797 | JN114627 | JN004521 |
| *O. equitans* | T. Motley & K. Cameron 2255 (NY) | AY907130 | AY907198 |
| *O. falconeri 1* | SBB-0793 | JN114632 | JN004526 |
| *O. falconeri 2* | SBB-0791 | JN114630 | JN004524 |
| *O. heliophila* | T. Motley & K. Cameron 2243 (NY) | AY907131 | AY907199 |
| *O. mucronata 1* | SBB-0232 | JN114640 | JN004534 |
| *O. mucronata 2* | SBB-0230 | JN114638 | JN004532 |
| *O. neocaledonic 1* | T. Motley & K. Cameron 2173 (NY) | AY907134 | AY907202 |
| *O. neocaledonic 2* | K. Cameron 27 (NY) | AY907133 | AY907201 |
| *O. pachyrachis 1* | SBB-0265 | JN114645 | JN004538 |
| *O. pachyrachis 2* | SBB-0263 | JN114643 | JN004536 |
| *O. pachyrachis 3* | SBB-0264 | JN114644 | JN004537 |
| *O. padangensis* | Leiden cult. 960231 | AY907135 | AY907203 |
| *O. setifera* | NYBG cult. s.n. ex Andy's Orchids | AY907136 | AY907204 |
| *O. wappeana* | Leiden cult. 20030243 | AY907138 | AY907206 |
